# Type II spiral ganglion afferent neurons drive medial olivocochlear reflex suppression of the cochlear amplifier

**DOI:** 10.1038/ncomms8115

**Published:** 2015-05-12

**Authors:** Kristina E. Froud, Ann Chi Yan Wong, Jennie M. E. Cederholm, Matthias Klugmann, Shaun L. Sandow, Jean-Pierre Julien, Allen F. Ryan, Gary D. Housley

**Affiliations:** 1 Translational Neuroscience Facility and Department of Physiology, School of Medical Sciences, UNSW Australia, Sydney, New South Wales 2052, Australia; 2 Inflammation and Healing Cluster, Faculty of Science, Health, Education and Engineering, University of the Sunshine Coast, Maroochydore, Queensland 4558, Australia; 3 Department of Psychiatry and Neuroscience, Laval University, Institut Universitaire en santé mentale de Québec, Quebec, Canada G1J2G3; 4 Departments of Surgery and Neurosciences, University of California San Diego, and Veterans Administration Medical Center, La Jolla, California 92093-0666, USA

## Abstract

The dynamic adjustment of hearing sensitivity and frequency selectivity is mediated by the medial olivocochlear efferent reflex, which suppresses the gain of the ‘cochlear amplifier' in each ear. Such efferent feedback is important for promoting discrimination of sounds in background noise, sound localization and protecting the cochleae from acoustic overstimulation. However, the sensory driver for the olivocochlear reflex is unknown. Here, we resolve this longstanding question using a mouse model null for the gene encoding the type III intermediate filament peripherin (*Prph)*. *Prph*^(−/−)^ mice lacked type II spiral ganglion neuron innervation of the outer hair cells, whereas innervation of the inner hair cells by type I spiral ganglion neurons was normal. Compared with *Prph*^(+/+)^ controls, both contralateral and ipsilateral olivocochlear efferent-mediated suppression of the cochlear amplifier were absent in *Prph*^(−/−)^ mice, demonstrating that outer hair cells and their type II afferents constitute the sensory drive for the olivocochlear efferent reflex.

## Abstract (web summary)

The medial olivocochlear efferent reflex regulates cochlear outer hair cell-based amplification of sound energy. Here the authors show this dynamic control of hearing sensitivity is driven by sensory input from the outer hair cells and their type II spiral ganglion neuron innervation.

## Graphical abstract

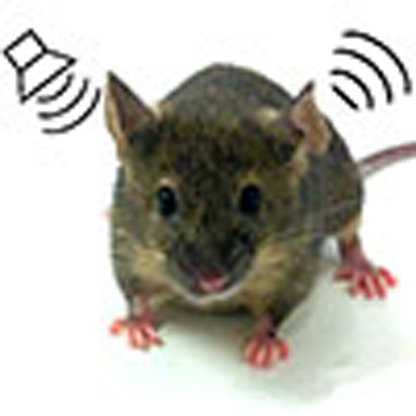

Hearing performance is dependent upon the active enhancement of sound-induced vibration of the cochlear organ of Corti through prestin-mediated outer hair cell (OHC) electromotility^1^. The action of this ‘cochlear amplifier'^2^,^3^ is responsible for the sensitivity and exquisite frequency resolution of mammalian hearing and can be assessed by measuring distortion product otoacoustic emissions (DPOAEs)^4^,^5^ in the ear canal. The cochlear amplifier selectively amplifies cochlear vibrations in a focused region of the cochlea, thereby enhancing sound transduction at inner hair cells (IHCs), each of which receives exclusive sensory innervation from several type I spiral ganglion neurons (SGNs), whose central projection to the brainstem cochlear nuclei drives hearing perception. The relative levels of sound transduced by IHCs between the two ears is principally regulated by a sensorimotor reflex pathway, which responds to elevation of sound in one ear by rapidly reducing the gain of the cochlear amplifier in the opposite cochlea^5^,^6^,^7^. This ‘contralateral suppression' is produced by medial olivocochlear (MOC) efferent neurons located in the superior olivary complex of the brainstem, whose myelinated axons approach the floor of the fourth ventricle from both sides before migrating laterally to join the ipsilateral auditory nerve and terminate on the OHCs^8^. These cholinergic MOC synapses hyperpolarize the OHCs, inhibiting OHC electromotility and thus reducing the gain of the cochlear amplifier^9^,^10^,^11^,^12^. In addition to contralateral suppression, there is also an ipsilateral pathway through which MOC neurons suppress the cochlear amplifier^7^,^13^. Efferent control of the cochlear amplifier has been shown to be necessary for speech discrimination in noise^14^,^15^, sound localization^16^ and protection from noise-induced hearing loss^17^. However, the sensory input that drives this neural control process has not been identified^8^.

The cochlear amplifier gain control reflex must either originate with primary auditory encoding of the sound perception channel by type I SGN, which make up 95% of the spiral ganglion, and/or with the residual 5% of the SGNs—designated type II, which exhibit a dispersed sensory innervation of OHCs^18^,^19^,^20^,^21^,^22^,^23^,^24^,^25^,^26^. The function of the type II SGN remains enigmatic^8^. Here, we assess the role of type I versus type II SGNs in the MOC reflex by utilizing a mouse model, null for gene encoding the type III intermediate filament peripherin (*Prph*^(−/−)^), that is found to lack type II SGN innervation of OHCs. Absence of suppression of the cochlear amplifier-derived DPOAEs in response to contralateral or ipsilateral sound in these *Prph*^(−/−)^ mice supports the hypothesis that the putative OHC—type II SGN sensory transmission drives the MOC efferent regulation of cochlear amplifier gain. Thus, MOC reflex control of hearing sensitivity utilizes a closed-loop negative-feedback pathway with the OHCs operating as both the sensor and effector.

## Results

### *Prph* ^(−/−)^ mice lack type II SGN innervation

In the mouse cochlea, peripherin (PRPH) is exclusive to the type II SGNs during the establishment and maturation of the OHC innervation^27^,^28^,^29^,^30^. We examined cochlear innervation patterns in an established *Prph*^(−/−)^ mouse line^31^ and found complete absence of OHC innervation by unmyelinated type II SGN neurites, consistent with key roles for PRPH in the regulation of peripheral sensory fibre extension and maintenance^28^,^32^,^33^,^34^. The *Prph*^(−/−)^ mouse line was initially characterized as lacking a substantial proportion of unmyelinated sensory fibres from the L5 dorsal root ganglion^31^, which prompted our assessment of this model to investigate the physiological significance of the unmyelinated cochlear type II SGN sensory fibres.

**Figure 1:**
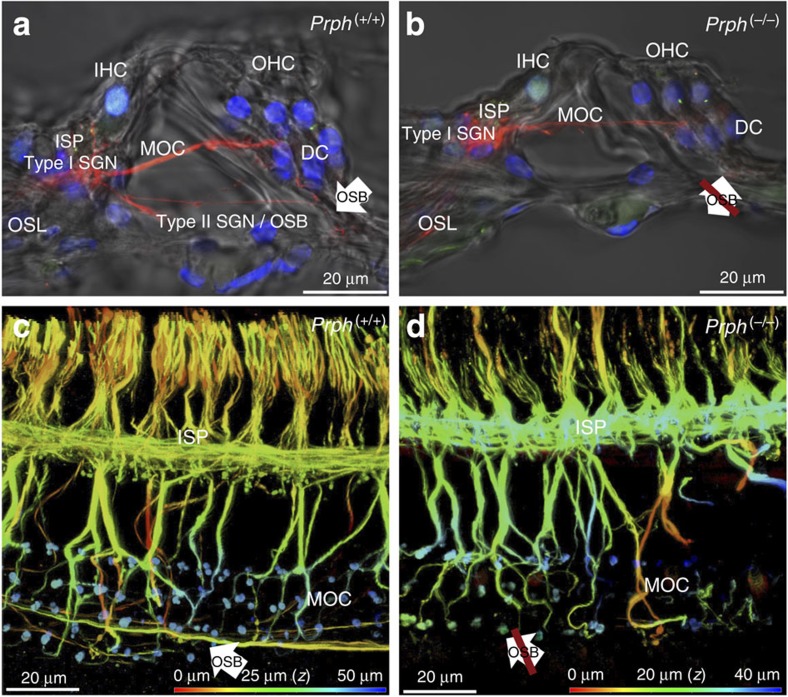
Loss of afferent innervation of outer hair cells (OHC) in *Prph*^(−/−)^ cochlea. (**a**) Type II spiral ganglion neuron (SGN) outer spiral bundle (OSB) innervation of the OHC runs alongside the Deiters' cells (DC) and is resolved in adult *Prph*^(+/+)^ organ of Corti via neurofilament 200 kDa immunofluorescence (red). This immunolabelling (confocal optical slice with transmitted light overlay) of a cryosection (50 μm) through the organ of Corti also identifies the medial olivocochlear (MOC) efferent fibres crossing the tunnel of Corti to innervate the OHC. CtBP2 immunofluorescence (green puncta) labels the presynaptic ribbons at the base of the OHC nuclei and at the inner spiral plexus (ISP) region of the inner hair cell (IHC) (the IHC nucleus is also labelled by this antibody). Blue fluorescence is DAPI nuclear staining. OSL, osseous spiral lamina. (**b**) Neurofilament 200 kDa immunofluorescence image of adult *Prph*^(−/−)^ organ of Corti in a cryosection, as for **a**, showing the absence of the type II SGN innervation of the OHC via the OSB. (**c**) The type II SGN OSB fibres were more fully resolved in the adult *Prph*^(+/+)^ organ of Corti mid-cochlear level whole mount via the neurofilament immunofluoresence (three-dimensional reconstruction of optical slices imaged from the basilar membrane; colour coded for depth (*z*)). Multiple fibres are shown crossing the floor of the tunnel of Corti, to enter the OSB and turn basally in parallel tracks (arrow). Deeper in the tissue, the MOC efferent fibres project to the OHC, terminating with bulbous synaptic boutons (VAChT immunolabelling; green–blue). (**d**) OSB fibres were absent in the adult *Prph*^(−/−)^ organ of Corti (crossed arrow); whole mount as for **c**. Note the retention of the MOC innervation to the OHC.

The type II SGN innervation of the OHCs was identified using PRPH immunofluorescence in cochlear cryosections and surface mounts of the *Prph*^(+/+)^ organ of Corti from postnatal day 4 (Supplementary Fig. 1). The PRPH-positive type II SGN neurites extend from the somata in Rosenthal's canal, past the type I SGN (radial fibre) innervation of the IHCs at the inner spiral plexus, crossing the floor of the tunnel of Corti. The type II SGN fibres then turn basally at the Deiters' cell level to form the outer spiral bundle (OSB) that innervates multiple OHCs^19^,^22^. This was most evident in the neonatal cochlea (Supplementary Fig. 1a,c–f), whereas PRPH immunolabelling in the adult cochlear type II SGN fibres diminished beyond the inner spiral plexus (Supplementary Fig. 1b,g,h), compared with resolution of the OSB with neurofilament 200 kDa (NF200) immunolabelling (Fig. 1a,c). The absence of PRPH in the *Prph*^(−/−)^ spiral ganglion was confirmed (Supplementary Fig. 1b right inset). The NF200 also resolved MOC efferent fibres, while co-immunolabelling with vesicular acetylcholine transporter (VAChT; ref. 17) showed the extension of the MOC fibres to clusters of efferent boutons at the base of the OHCs (Fig. 1a–d and Supplementary Movies 1 and 2).

The OSB was absent in the *Prph*^(−/−)^ organ of Corti (Fig. 1b,d), a few residual type II SGN fibres were occasionally observed extending toward the OHCs, although none are visible in the fields shown here. In contrast, the MOC efferent innervation of the OHCs was preserved (Fig. 1b,d and Supplementary Movie 2). The loss of the OSB in the *Prph*^(−/−)^ cochleae was confirmed in eight adult mice, alongside well-resolved OSBs imaged in cochleae from five *Prph*^(+/+)^ and one *Prph*^(+/−)^ animal, using NF200 immunofluorescence. Thus knockout of *Prph* expression selectively disrupted the type II SGN afferent innervation of the OHCs.

Serial block face imaging in the (field emission) scanning electron microscope (SBF-SEM) resolved the small type II SGN afferent synaptic boutons adjacent to the large MOC efferent boutons at the base of the *Prph*^(+/+)^ OHCs in the mid-apical turn region of the cochlea (Supplementary Fig. 2a,c,d,g). No type II SGN synaptic boutons were identified in *Prph*^(−/−)^ OHCs (Supplementary Fig. 2b,e,f,g). The *Prph*^(+/+)^ OHCs typically had a single type II SGN bouton with a mean volume of 1.20±0.10 μm^3^ (average of data from three blocks from different mice), as well as three MOC efferent boutons (mean volume=14.22±0.83 μm^3^), consistent with data from conventional transmission electron microscopy (TEM) analysis, including discrimination of the efferent synapses by their size, abundant mitochondria and typical presence of electron-dense postsynaptic cisternae^35^. In the *Prph*^(−/−)^, the efferent bouton number and distribution was comparable to the *Prph*^(+/+)^ OHCs, with some hypertrophy (mean volume 19.05±0.95 μm^3^ (*n*=3); *P*=0.0186 (SA1)) (Supplementary Fig. 2g; Supplementary Table 1).

### *Prph* ^(−/−)^ mice lack contralateral suppression

**Figure 2:**
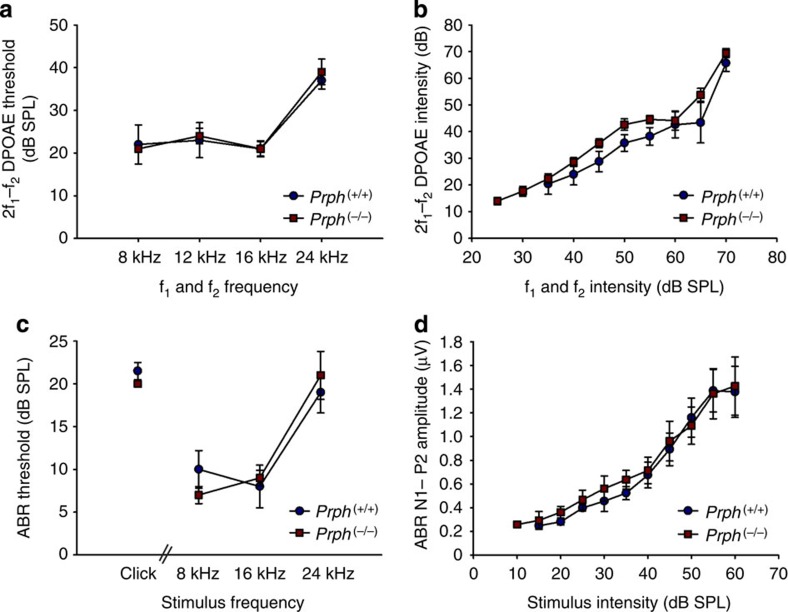
*Prph*^(−/−)^ mice have baseline hearing indistinguishable from *Prph*^(+/+)^ littermate mice. (**a**,**b**) Cubic (2f_1_–f_2_) distortion product otoacoustic emission (DPOAE) thresholds and 8 kHz input–output functions. (**c**,**d**) Auditory brainstem response (ABR) threshold and 8 kHz input–output functions for the N1-P2 wave, which reflects cochlear nerve recruitment (*n*=5). Data are shown as mean±s.e.m.; *P*>0.05 (SA8).

Baseline hearing function was indistinguishable between the *Prph*^(−/−)^ and *Prph*^(+/+)^ mice, as assessed using threshold and input–output functions (ref. 36) for both auditory brainstem responses (ABR) and cubic (2f_1_–f_2_) DPOAEs (Fig. 2).

**Figure 3:**
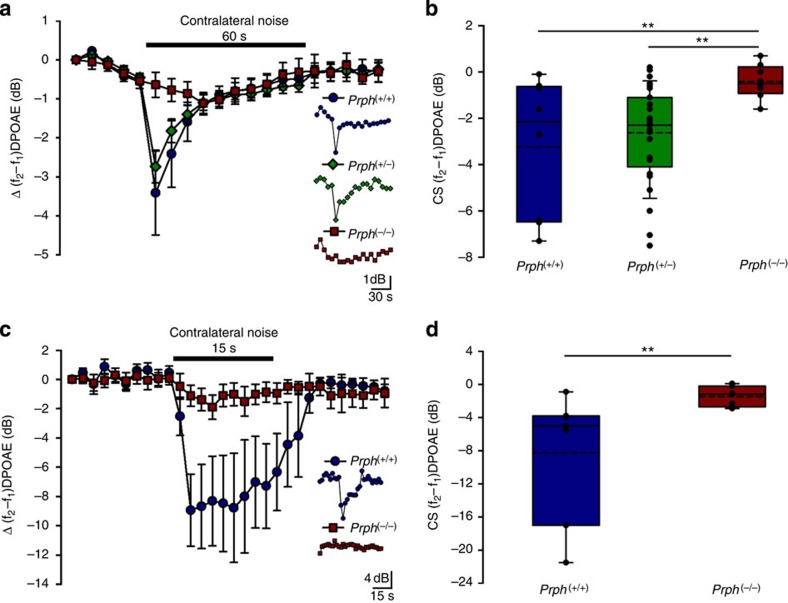
*Prph*^(−/−)^ mice lack contralateral suppression. (**a**,**b**) Contralateral suppression was observed as a significant reduction in the amplitude of the ipsilateral f_2_–f_1_ DPOAE (65 dB about 28 kHz) in *Prph*^(+/+)^ and *Prph*^(+/−)^ littermates in response to 82 dB 10–17 kHz contralateral sound (60 s). In contrast, rapid contralateral suppression was absent in *Prph*^(−/−)^ littermate mice (**P=0.007 *Prph*^(+/+)^ versus *Prph*^(−/−)^, *P*=0.002 *Prph*^(+/−)^ versus *Prph*^(−/−)^, (SA9); *n*=8 for (+/+) and (−/−), *n*=25 for (+/−)). Data in **b** are from the first measurement after noise onset relative to the average of the pre-noise baseline. (**c**,**d**) In a separate set of experiments with contralateral suppressor 96 dB 15–25 kHz and 60 dB about 20 kHz for the DPOAE driver, *Prph*^(+/+)^ mice had higher rapid-onset suppression, whereas the *Prph*^(−/−)^ mice showed a small response with slow kinetics (**P=0.011 (SA5), *n*=7 for each). Data in **d** are the average of the nine measurements during the 15 s contralateral noise, relative to the average of the pre-noise baseline. Insets in **a** and **c** show example recordings. Box plots in **b** and **d** have boundaries indicating 25th and 75th percentile, solid line is median, dashed line is mean, with individual data overlaid; error bars indicate 95% confidence limits. For **a** and **c**, data are shown as mean±s.e.m.

The functional effect of *Prph* gene deletion on contralateral-evoked MOC suppression was investigated using quadratic (f_2_–f_1_) DPOAEs, a measure of cochlear amplifier gain that is modulated by MOC efferent drive^5^. The contralateral suppression protocol was initially optimized for *Prph*^(+/+)^ mice (Supplementary Fig. 3) and three separate trials were then performed using a range of stimuli delivered to the contralateral ear, with DPOAEs recorded from the ipsilateral ear. *Prph*^(+/+)^, *Prph*^(+/−)^ and *Prph*^(−/−)^ littermates from heterozygous breeding pairs were used in a masked study with post-measurement genotyping. Two unmasked studies utilized homozygous *Prph*^(−/−)^ mice, with age- and sex-matched wild types from the background strain (129Sv/C57BL/6). In the masked study, *Prph*^(+/+)^ mice (*n*=8) showed a mean reduction of the ipsilateral DPOAE amplitude of 3.2±1.1 dB in response to an 82 dB SPL 10–17 kHz contralateral sound (60 s continuous) (Fig. 3a,b). This effect was evident from the onset of the contralateral sound, with maximal suppression of the OHC cochlear amplifier occurring within 5 s (Fig. 3a). The suppression exhibited near-complete adaptation at 30 s sound presentation. Heterozygous (*Prph*^(+/−)^) littermates showed comparable contralateral suppression (2.6±0.4 dB, *n*=25) with adaptation. In contrast, *Prph*^(−/−)^ littermates exhibited a significant lack of contralateral suppression, with no distinguishable adaptation at the peak response time of the wild-type and heterozygous mice (0.4±0.3 dB (*n*=8); (*Prph*^(+/+)^ versus *Prph*^(−/−)^ *P*=0.007, *Prph*^(+/−)^ versus *Prph*^(−/−)^ *P*=0.002 (SA2); Fig. 3a,b). The maximum residual reduction in quadratic DPOAE with contralateral noise for *Prph*^(−/−)^ mice occurred at ∼30 s (1.1±0.3 dB; *P*=0.008 (SA3)), merging with the residual adapting responses evident in the *Prph*^(+/+)^ and *Prph*^(+/−)^ mice. Average starting f_2_–f_1_ DPOAE amplitudes were 29.4±1.5 dB (+/+), 26.5±1.4 dB (+/−) and 18.0±2.7 dB (−/−).

The first unmasked study used a shorter duration, more intense and wider band contralateral sound exposure (96 dB SPL, 15–25 kHz) than that used for the masked study (Fig. 3c,d). For these stimulus conditions, *Prph*^(+/+)^ mice showed greater contralateral suppression (average over 15 s=8.2±2.9 dB, *n*=7), with the peak suppression occurring ∼3 s after contralateral noise onset. The *Prph*^(−/−)^ mice exhibited a slower rate of change in their DPOAEs, with an average suppression of 1.5±0.4 dB (*P*=0.0172 (SA4), *n*=7). Adaptation of the contralateral suppression was negligible with this duration of contralateral sound (*P*=0.011 (SA5) comparing the average of the contralateral suppression in *Prph*^(−/−)^ and *Prph*^(+/+)^). Baseline f_2_–f_1_ DPOAE amplitudes were 17.2±2.8 (+/+) and 17.4±2.1 (−/−). This result was independently replicated in a second unmasked study, where contralateral noise duration was varied (5 to 30 s) to probe the absence of rapid-onset contralateral suppression in the *Prph*^(−/−)^ mice (Supplementary Fig. 4). These three independent experiments demonstrated that *Prph*^(−/−)^ mice lack the MOC-mediated contralateral suppression observed in wild types.

We validated our contralateral suppression model in two control studies: acoustic cross-talk between cochleae was excluded by physically disrupting the contralateral tympanic membrane and ossicles (contralateral suppression 5.5±0.9 dB before versus 0.2±0.2 dB after; *P*=0.004 (SA6), *n*=3; 30 s 82 dB SPL 10–17 kHz noise; primaries at 65 dB SPL ∼28 kHz). To confirm that the MOC efferent pathway was involved (and eliminate a role for the middle ear muscle reflex, which drives the ossicular chain tensor tympani and stapedius muscles, increasing middle ear impedance and reducing sound transmission during loud sound and vocalization^37^), we made a rostral–caudal incision at the floor of the fourth ventricle (Supplementary Fig. 5, contralateral suppression 3.9±0.6 dB before versus 1.2±0.3 dB post section, *P*=0.043 (SA6), *n*=3; ref. 7).

### *Prph* ^(−/−)^ mice lack ipsilateral suppression

Ipsilateral suppression is stronger than contralateral suppression, but more challenging to quantify because the sound used to elicit the MOC reflex is delivered over both the primary tones and the DPOAEs^13^. We established a mouse model for ipsilateral suppression of the cochlear amplifier by measuring the recovery from a brief ipsilateral noise during on-going quadratic DPOAE measurement.

**Figure 4:**
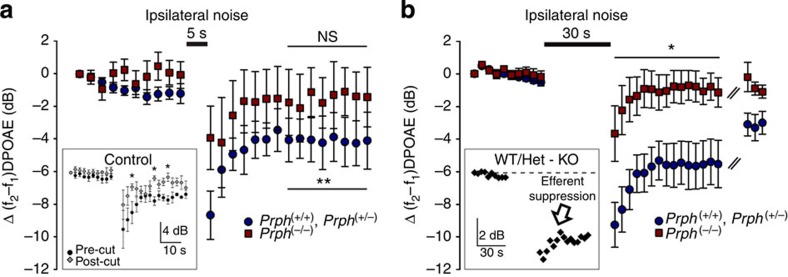
*Prph*^(−/−)^ mice lack ipsilateral suppression. (**a**) 5 s of 82 dB SPL 10–25 kHz ipsilateral suppressor sound produced a sustained reduction in the amplitude of the ipsilateral f_2_–f_1_ DPOAE (60 dB SPL ∼20 kHz) in *Prph*^(+/+)^ and *Prph*^(+/−)^ mice (**P=0.004 (SA3), 21–45 s post noise (lower bar, comparing mean values); *n*=9). In contrast, *Prph*^(−/−)^ mice had only a small transient modulation (upper bar; *P*=0.461 (SA4); *n*=6). Inset in **a** is a control experiment in *Prph*^(+/+)^ mice that confirmed the involvement of the MOC reflex by sectioning the olivocochlear bundle at the floor of the fourth ventricle (*P=0.049, 0.022, 0.028 for sequential pre- versus post-cut comparisons as indicated (SA7), *n*=3). (**b**) Comparison of 30 s of 82 dB 15–25 kHz ipsilateral suppressor sound on f_2_–f_1_ DPOAE (60 dB SPL ∼20 kHz) in *Prph*^(+/+)^ and *Prph*^(+/−)^ versus *Prph*^(−/−)^ mice (*P=0.032 (SA7), *n*=17 for (+/+)/(+/−), *n*=6 for (−/−)). Breaks in the data indicate a 6 min interval. Inset in **b** extracts the OHC—type II SGN—MOC efferent reflex from the initial co-incident off-sound transient (WT/HET—KO). Data are shown as mean±s.e.m.

MOC-recruiting ipsilateral sound stimulation (82 dB SPL, 10–25 kHz, 5 s) was presented over the DPOAE primary tones (60 dB SPL, around 20 kHz). Immediately after the suppressor noise ended, DPOAE measurements were restored and a pronounced reduction in quadratic DPOAE was evident in *Prph*^(+/+)^ and *Prph*^(+/−)^ mice. This ipsilateral suppression adapted within ∼10 s, to a sustained offset from the pre-noise intensity (immediate DPOAE intensity reduction 9.7±2 dB; average DPOAE reduction at 21 to 45 s post noise 4±1.4 dB; *P*=0.004 (SA3); *n*=9; Fig. 4a). These results contrasted with a smaller immediate DPOAE reduction post noise (5.0±1.9 dB) in the *Prph*^(−/−)^ mice, that very rapidly (∼10 s) returned to baseline (1.4±1.7 dB; *n*=6; *P*=0.461 (SA4); Fig. 4a). Average starting f_2_–f_1_ DPOAE amplitudes were 15.5±2.0 dB (+/+)/(+/−) and 10.3±0.8 dB (−/−). The role of the ipsilateral MOC reflex in this response was confirmed by section of the MOC pathway at the floor of the fourth ventricle in *Prph*^(+/+)^ and *Prph*^(+/−)^ mice (Fig. 4a inset; *n*=3). This showed that the immediate post noise reduction in DPOAE was reduced to that seen in the *Prph*^(−/−)^ mice, and the measurements from ∼9 s post noise similarly returned to baseline. Thus ipsilateral suppression without the MOC contribution in *Prph*^(+/+)^ mice was indistinguishable from that observed in *Prph*^(−/−)^ mice with an intact MOC fibre tract.

In a second set of experiments with a different group of mice, 30 s of 82 dB SPL, 15–25 kHz ipsilateral sound was presented to drive ipsilateral suppression (Fig. 4b). In *Prph*^(+/+)^and *Prph*^(+/−)^ mice, the magnitude of the off-noise transient reduction in the quadratic DPOAE was comparable to that seen after 5 s of ipsilateral stimulation (immediate reduction 10.6±1.5 dB; average DPOAE reduction at 21 to 45 s post noise 5.1±1.4 dB). Again, no sustained modulation was seen in the *Prph*^(−/−)^ mice (immediate reduction 4.4±1.6 dB; average DPOAE reduction at 21 to 45 s post noise 0.7±0.9 dB; *P*=0.032 (SA7), 21 to 45 s post noise; *n*=17 for (+/+)/(+/−), *n*=6 for (−/−)). Average starting f_2_–f_1_ DPOAE amplitudes before noise were 17.5±1.0 dB (+/+)/(+/−) and 15.2±1.6 dB (−/−). These data were used to extract the efferent response from the immediate off-noise transient (*Prph*^(+/+)/(+/−)^ minus *Prph*^(−/−)^ indicated as ‘WT/Het—KO', Fig. 4b inset), which remains after cutting the MOC tract in WT mice (Fig. 4a inset). The latter is likely to reflect transient noise-induced changes in electrochemical driving force^38^. This analysis revealed MOC-mediated ipsilateral suppression, which adapted over ∼21 s to a residual offset that lasted for several minutes (see data after breaks in Fig. 4b showing recovery).

## Discussion

**Figure 5:**
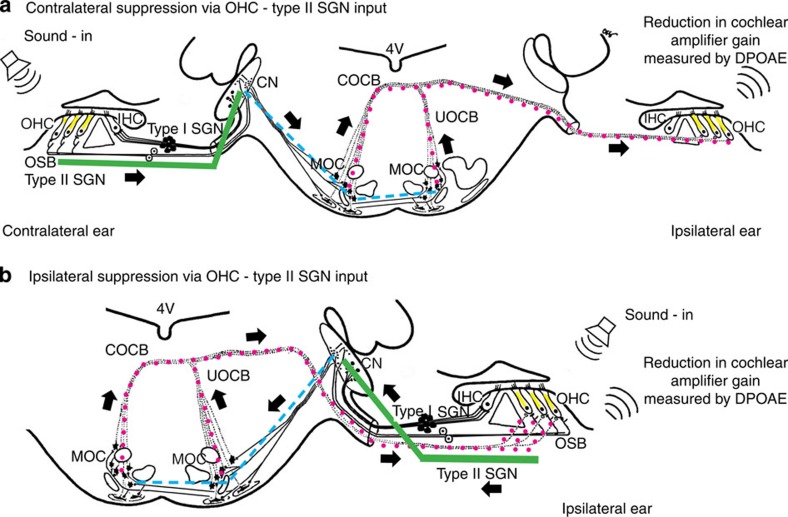
Schematic of the sensorimotor reflex pathways for contralateral and ipsilateral suppression of the cochlear amplifier that provides dynamic control of the sensitivity and frequency selectivity of hearing. The schematic includes the outer hair cell (OHC)—type II spiral ganglion (SGN) sensory input described here, with the previously established medial olivocochlear (MOC) efferent reflex pathway (derived from refs 21, 54, 55; note that in the mouse, ∼75% of the medial olivocochlear (MOC) efferent fibres are within the crossed olivocochlear bundle (COCB; reviewed by ref. 56). (**a**) Contralateral suppression: sound in one ear causes a reduction in distortion product otoacoustic emissions (DPOAE) in the opposite ear, reflecting a reduction in the gain of the OHC-based cochlear amplifier. Type II spiral ganglion neuron (SGN) afferent fibres (green line) project to the cochlear nuclei (CN). A relay neuron pathway (blue dashes) then drives the MOC neurons located in the superior olivary complex on both sides of the brainstem. These MOC neurons project axons via crossed and uncrossed (UOCB) tracts (red dots) that approach the floor of the fourth ventricle (4 V), before joining the cochlear/vestibular nerve. These MOC efferent fibres terminate as cholinergic boutons at the base of the OHCs. In contrast, the sensory innervation of the inner hair cells (IHC) by the type I SGN, which drive hearing perception, do not contribute to this reflex regulation of the cochlear amplifier. (**b**) Ipsilateral suppression: noise in one ear can suppress the DPOAE in the same ear via the OHC—type II SGN—MOC reflex pathway.

Our findings determine that the OHC—type II SGN sensory pathway drives the MOC efferent reflex-mediated control of the cochlear amplifier. Thus, not only does sound transduction by OHCs produce the electromotility that is the substrate of the cochlear amplifier, but integration of OHC transduction at the type II SGN afferent synapses provides the sensory coding of cochlear amplifier gain that drives the negative feedback control via the MOC efferent innervation to both cochleae (evident as contralateral (Fig. 5a) and ipsilateral (Fig. 5b) suppression).

No functional contribution to hearing has previously been established for type II SGN^8^,^25^; although central projections to the cochlear nuclei alongside the type I SGN have been documented^25^,^39^. Type II SGN excitability has been demonstrated *in vivo* by cochlear electrical stimulation^40^, and their membrane properties demonstrated *in vitro*^22^,^23^,^24^. Significantly, type II SGN make multiple *en passant* synapses across multiple rows of OHCs approximately a quarter of an octave basal to the corresponding innervation of IHC by the type I SGN radial fibres^8^,^25^. This is consistent with the coding of the active region of the cochlear amplifier relative to the tonotopic coding by the corresponding IHC^8^. The match of the type II SGN OHC innervation to the MOC efferent regulation of the OHC-controlled cochlear biomechanics was originally highlighted by Kim^20^. The concept has also been supported by the recent electrophysiological evidence from isolated rat organ of Corti, showing integration of synaptic input from multiple OHCs by type II SGN^26^.

The role of type II SGN fibres in conveying the operational state of the cochlear amplifier to the MOC neurons is also compatible with the known central projections of these fibres to the cochlear nuclei. The type II SGN input overlaps extensively with the type I SGN projections in the dorsal and ventral nuclei, as well as having differential input to the granule cell region (reviewed by Nayagam *et al*.^25^). Transneuronal retrograde labelling via pseudorabies virus injections into the cochlea indicate that the cochlear sensory drive to the MOC efferent neurons within the superior olivary complex is most likely relayed through planar multipolar cells of the posterior and anterior ventral cochlear nuclei^41^, which overlaps with the type II SGN projection.

The quadratic DPOAE contralateral suppression adaptation profile, which takes tens of seconds, is consistent with slow adaptation of MOC efferent-mediated inhibition of the cochlear amplifier downstream of the OHC α9/α10 nAChRs^42^,^43^,^44^. Our *Prph*^(−/−)^ model has also enabled the isolation of the sensory drive for MOC efferent suppression arising from ipsilateral sound. These data show that the ipsilateral suppression is substantially stronger than the contralateral suppression for the same input intensity, and adapts more slowly than the contralateral suppression. This is evident from the sustained ∼5 dB suppression after 30 s of ipsilateral noise (82 dB SPL; Fig. 4b,b inset) compared with <1 dB residual suppression after 30 s contralateral noise (82 dB SPL; Fig. 3a). Consequently, the rapid adaptation of contralateral suppression likely reflects ipsilateral adaptation of the sensory coding from the cochlear amplifier via the OHC—type II SGN in the (contralateral) cochlea receiving the noise probe, rather than adaptation of the downstream MOC efferent output to the opposite cochlea. Hence, the sensory drive for contralateral suppression attenuates rapidly. The dominance of ipsilateral suppression is correlated with the bias in neural connectivity from the ipsilateral cochlear nuclei to the MOC neurons on the opposite side of the brainstem^41^. The discrimination between ipsilateral and contralateral suppression properties achieved here by isolating the type II fibre sensory driver has considerable significance for understanding the weighting and differential adaptation of the gain control of the cochlear amplifiers in each ear, and is likely to have bearing on the design of cochlear implant systems for binaural implantation.

Our study is also informative with regard to the role of the intermediate filament PRPH, in sustaining neural connectivity. Several studies have demonstrated its action in other neural populations, including the finding that a subpopulation of unmyelinated dorsal root ganglion sensory neurons was absent in the *Prph*^(−/−)^ mouse^31^.

Atrophy of peripheral neurites in the type II SGN OSB fibres was nearly complete in the *Prph*^(−/−)^ mice. This is likely due to loss of PRPH-dependent neurite extension and stabilization cues^33^ in the critical postnatal period for establishment of the cochlear afferent innervation^25^,^27^,^29^. In this regard, the mouse model provided a fortuitous advantage, as *Prph* expression by SGN is constrained to the type II fibres from around E18 in the mouse, which precedes the afferent innervation of the hair cells^27^,^29^,^30^. In the rat, *Prph* expression is sustained in the type I SGN over the early critical afferent innervation period^34^,^45^. It has not been established whether mouse type I SGN fibres express *Prph* at earlier embryonic periods, but the normality of the ABR function in the *Prph*^(−/−)^ mice indicates that, even if this is the case, it has not affected the establishment of the central auditory circuitry, making it unlikely that an indirect action on type I fibres is a factor in the loss of the MOC reflex suppression of outer hair cell electromotility evident in these mice. Peripherin expression in the adult mouse brainstem is highly constrained and, outside of the central projections of the type II SGN, is not correlated with the MOC reflex pathway^46^.

The elimination of the type II afferent boutons may also have effects on the local neural circuitry within the organ of Corti, as some interconnectivity between type II afferent fibres, MOC efferent varicosities and inner hair cells has been identified^47^. The hypertrophy of the MOC efferent boutons at the base of the OHCs in the *Prph*^(−/−)^ mice is consistent with the reduction in sensory drive^48^ to the central MOC efferent reflex circuit, evident as a loss of dynamic suppression of the DPOAEs when sound is presented to the ears.

Our data are consistent with the concept that the binaural regulation of hearing sensitivity via the MOC efferent fibre-mediated suppression of cochlear amplifier gain is largely an autonomous reflex that utilizes a putative non-perceptive auditory coding channel provided by the OHCs and type II SGNs. This complements the lateral superior olivary complex efferent fibres, which directly regulate the type I SGN output via presynaptic termination beneath the IHCs^12^,^49^. The integrated encoding of OHC transduction that underpins the cochlear amplifier state, provides the sensory drive to adjust gain between the two ears. This is evidently critical for balanced hearing, which enables optimal sound localization^16^ and speech discrimination in noise^14^,^15^,^50^. The MOC efferent pathway has also been implicated in protection from hearing loss from sustained noise^17^. Given that type II SGN loss is prevalent with aging^51^, degradation of the OHC/type II SGN drive to MOC efferent-based suppression of the cochlear amplifier may contribute to the decline in hearing performance in noise with aging, and to age-related hearing loss.

## Methods

### Animals

Male and female adult CBA/129 wild-type, 129Sv/C57BL/6 wild-type and *Prph*^(−/−)^ mice on a 129Sv/C57BL/6 background were used for this study. The *Prph*^(−/−)^ mouse model was established in 2001 using 129Sv strain embryonic stem cells bearing the peripherin knockout construct (exon 1 deletion) that were injected into C57BL/6 strain blastocysts. The chimeric mice were then crossed with C57BL/6 mice to produce heterozygous peripherin KO mice^31^. In masked studies, heterozygous breeders were used to provide littermates of identical genetic background. PCR-based genotyping utilized the following primer sets: Prph-5′-UTR-F: 5′-GCTCCTTGCCACCCGGCCTAGTTC-3′; Prph-exon1-R: 5′-AGGGCTGCGTTCTGCTGCTC-3′; Neo-F: 5′-TTCGGCTATGACTGGGCACAACAG-3′; Neo-R: 5′-TACTTTCTCGGCAGGAGCAAGGTG-3′. Immunolabelling with a peripherin antibody (see Immunohistochemistry—below) validated the loss of peripherin protein in the *Prph*^(−/−)^ mice. Experiments were conducted according to UNSW Australia Animal Care and Ethics Committee guidelines.

### Hearing function tests

Mice were anaesthetized using a ketamine cocktail containing ketamine (40 mg kg^−1^), xylazine (8 mg kg^−1^) and acepromazine (0.5 mg kg^−1^) via intraperitoneal injections. Hearing testing was carried out using an auditory-evoked potential and DPOAE workstation (TDT system 3 with RX6 and RX6–2 signal processors, Tucker Davis Technologies, Ft Lauderdale, FL, USA) with BioSig32 software. Sound levels were calibrated using a one-quarter-inch Free Field Measure Calibration Microphone (model 7016; ACO). Hearing testing was carried out in a sound-attenuating chamber (Sonora Technology, Japan). The level of anaesthesia (indicated by heart rate and breathing rate) was monitored using a mouse oximetry system (MouseOx, STARR Life Sciences) and depth of anaesthesia was further assessed by lack of tail-pinch response. The core body temperature of the mice was clamped at 37 °C by feedback control of a heating pad (Right Temp, Able Scientific).

DPOAE: The right external auditory meatus of each mouse was coupled to a small microphone used to detect changes in sound pressure which represent DPOAEs (Etymotic ERB10+, Etymotic Research). Two EC1 electrostatic speakers, controlled by the Tucker Davis Technologies (TDT) system (BioSigRP software), generated equal intensity primary tones (f_1_ and f_2_; f_2_/f_1_ ratio: 1.25). Recordings were taken at 8, 12, 16 and 24 kHz presented from 0 to 70 dB SPL (increasing in 5 dB steps). Fifty measurements at 6.7 per second were averaged for each stimulus intensity and frequency, which were analysed by Fast Fourier transformation. Thresholds at the different frequencies were determined based on when the cubic (2f_1_–f_2_) distortion product reached 5 dB above the noise floor, as the tone intensity increased.

ABRs were recorded using three platinum subdermal electrodes inserted subcutaneously at the vertex (+), over the mastoid process (−) and near the base of the tail (ground). An EC1 speaker provided either click, or puretone pip (8, 16 and 24 kHz) stimuli at 10 per second, in descending 5 dB steps (starting at 70 to 80 dB SPL). Each trace studied is an average of up to 512 raw recordings at each intensity and frequency. Thresholds at the different frequencies were determined as the lowest intensity at which ABR waves could be observed.

MOC efferent-mediated suppression of DPOAE: Either the contralateral or ipsilateral ear was exposed to broadband suppressor sound stimulation while DPOAEs were elicited using primary tones in the ipsilateral ear. 10–50 measurements were averaged (6.7/s) for each recording. Baseline DPOAE measurements were taken before and recovery was monitored following the suppressor stimulus. The amplitude of the quadratic (f_2_–f_1_) distortion product relative to the noise floor was then analysed before, during and after the noise stimulation. Broadband noise was generated using a National Instruments A/D, with custom software to create the noise. The output was amplified using an SA1 amplifier (TDT) and delivered closed-field via either an MF1 or FF1 speaker (TDT) using a coupler.

### Surgical disruption of MOC efferent activity

These were all carried out in adult CBA/129mice under ketamine (40 mg kg^−1^), xylazine (8 mg kg^−1^) and acepromazine (0.5 mg kg^−1^) anaesthesia. The contralateral tympanic membrane and ossicles were surgically disrupted. Sectioning of the MOC neurons: The contralateral suppression was robust and repeatable after an hour under the ketamine/xylazine/acepromazine anaesthesia. Subsequently the MOC efferent fibres beneath the floor of the fourth ventricle were sectioned with a scalpel blade incision just ipsilateral to the midline, achieved via a dorsal fenestration between C1 and the foramen, with supplementary isoflurane anaesthesia.

### Immunohistochemistry

Immunohistochemical labelling was carried out on fresh tissue from unexposed animals. Mouse cochleae were fixed by scali perfusion of 4% paraformaldehyde, decalcified for 14 days in 8% EDTA and then dissected for surface mount preparation or cryoprotected using sucrose and OCT before being cryosectioned at 50 μm. Nonspecific binding was blocked with 10–15% normal goat or donkey serum, 1% Triton X-100 in phosphate-buffered saline (PBS) for 1 h at room temperature (RT) and sections were then transferred to the primary antibody solution; Neurofilament 200 kDa (Sigma, rabbit, 1:5,000); peripherin (EB12405, Everest Biotech, goat, 1:10,000); CtBP2 (BD Bioscience, mouse, 1:500); vesicular acetylcholine transporter (VAChT; Phoenix, goat, 1:200) in 5–15% normal goat or donkey serum, 0.1% Triton X-100 in PBS) overnight at RT. The sections were washed in PBS and appropriate secondary antibody was applied overnight RT (Molecular Probes, 1:500 anti-rabbit IgG AlexaFluor 594, anti-goat IgG AlexaFluor 488, anti-mouse IgG AlexaFluor 488). Following a further PBS wash, DAPI was applied for 5 min at 1:5,000. Two final washes in PBS were carried out before the tissue was mounted using Vectashield (Vector Laboratories) and imaged on a Zeiss confocal microscope (Zeiss 710 NLO). Data were obtained from 1 to 3 organ of Corti z stacks (30 μm) analysed per animal.

### SBF-SEM*—*tissue preparation

Perfusion fixed cochleae were processed for 3View (Gatan) field emission scanning electron microscopy (FESEM, Zeiss Sigma) incorporating sequential *en bloc* staining. Fixation included perfusion, after flushing, with 1% paraformaldehyde, 3% glutaraldehyde in 0.1 M sodium cacodylate buffer, with 0.1 M sucrose, 10 mM betaine, 2 mM CaCl_2_ (CSBC buffer), pH 7.4 for 1–2 h. Cochleae were then dissected, small windows cut in the labyrinth wall and further perfusion fixed in the above solution. With continual light agitation on a rotor, tissue was subsequently processed through successive solutions of; CSBC buffer (3 × 5 min), 3% K_3_Fe(CN)_6_ in CSBC buffer, 1:1 with 4% OsO_4_ (1 h, 4 °C), CSBC buffer (3 × 5 min), 1% tannic acid in CSBC buffer (1 h), CSBC buffer (3 × 5 min), 0.1% thiocarbohydrazide (20 min), MQ water (3 × 5 min), 4% OsO_4_ (aqueous; 30 min), MQ water (3 × 5 min), saturated uranyl acetate (aqueous; 4 °C, 18 h), MQ water (3 × 5 min), lead citrate (30 min, 60 °C), MQ water (3 × 5 min) and dehydrated in an alcohol–acetone series for subsequent embedding in Araldite 502.

Conventional TEM was used to identify tissue preparation quality, and the outer hair cell region, with samples imaged at x1–40 k using a digital TEM camera at 4 MP. Block faces were then trimmed to ∼50–80 μm^2^ to facilitate optimal imaging for subsequent SBF-SEM (3View, Gatan). Serial 50 nm sections were cut and block faces imaged through ∼50 μm of tissue per block. Blocks from three mice per genotype were scanned. Serial section EM, along with the increased electron density of the afferent boutons and evident synaptic vesicles and an electron-dense postsynaptic cistern in the efferent boutons provided unequivocal discrimination of the type II afferent boutons from the MOC efferent boutons.

Outer hair cells and their associated afferent and efferent synaptic boutons were manually identified from their characteristic morphology, and segmented using TrakEM2 (ref. 52), a plugin developed for ImageJ (NIH) and packaged in the FIJI distribution^53^. Segmentation of multiple slices along the axial plane yielded three-dimensional reconstructions on which volume measurements were made.

### Statistical analysis

Data are expressed as population mean±s.e.m. Statistical analysis was carried out in SigmaPlot (V. 11, Systat Software, Germany). Statistical tests cited in the text are indicated in Table 1. Holm–Sidak *post hoc* analysis was utilized for multiple pairwise comparisons within analysis of variance, providing a correction for repeated testing to maintain the type I error rate (false positive). Data were tested for normal data distribution and a rank transformation was applied where required. Power calculations based on pilot experiments informed experimental design.

## Author contributions

G.D.H. and A.F.R. conceived and designed the project. K.E.F., A.C.Y.W., J.M.E.C. and G.D.H. performed experiments and analysed the data, performed immunolabelling analysis with contributions from M.K. S.L.S., K.E.F. and G.D.H. undertook the FESEM, with S.L.S. performing the serial reconstructions of the OHC synaptic boutons. G.D.H., K.E.F. and A.F.R. wrote the manuscript with significant input from the other contributors. J.-P.J. provided materials and support for the peripherin knockout mouse model.

## Additional information

**How to cite this article:** Froud, K. E. *et al*. Type II spiral ganglion afferent neurons drive medial olivocochlear reflex suppression of the cochlear amplifier. *Nat. Commun*. 6:7115 doi: 10.1038/ncomms8115 (2015).

## Supplementary Material

Supplementary Information Supplementary Figures 1-5, Supplementary Table 1, and Supplementary References

Supplementary Movie 1 Immunofluorescence imaging of the afferent and efferent innervation in Prph(+/+) organ of Corti using Neurofilament 200kDa and VAChT. 3D rendering of confocal z stack from the basilar membrane with depth coding. Red is closest to the basilar membrane – isolating the type I SGN innervation within the inner spiral plexus and the type II SGN outer spiral bundle fibres that run basal to the three rows of outer hair cells; blue is deepest and resolves the medial olivocochlear efferent innervation, including the associated VAChT immunolabelled efferent boutons synapsing at the base of the outer hair cells (refer to Fig. 1c for details).

Supplementary Movie 2 Immunofluorescence imaging of the afferent and efferent innervation in Prph(-/-) organ of Corti using Neurofilament 200kDa and VAChT. 3D rendering of confocal z stack from the basilar membrane with depth coding. Red is closest to the basilar membrane – isolating the type I SGN innervation within the inner spiral plexus; note the absence of type II SGN outer spiral bundle fibres that in Prph(+/+) mice run basal to the three rows of outer hair cells. Blue is deepest and resolves the medial olivocochlear efferent innervation, including the associated VAChT immunolabelled efferent boutons synapsing at the base of the outer hair cells (refer to Fig. 1d for details).

## Figures and Tables

**Table 1:** Statistical tests.

Code	Statistical procedure
SA1	Two-tailed *t*-test
SA2	One-way ANOVA
SA3	Single-sample signed rank test
SA4	Single-sample *t*-test (two tailed)
SA5	Mann–Whitney rank-sum test
SA6	One-way repeated measures ANOVA
SA7	Two-way repeated measures ANOVA on ranked data
SA8	Two-way repeated measures ANOVA
SA9	One-way ANOVA on ranked data

ANOVA, analysis of variance.
