# Supplementary material for: Type II spiral ganglion afferent neurons drive medial olivocochlear reflex suppression of the cochlear amplifier
Source: Nat Commun. 2015 May 12;6:7115. doi: 10.1038/ncomms8115 (PMC4432632; doi:10.1038/ncomms8115)
Supplement: Supplementary Information — Supplementary Figures 1-5, Supplementary Table 1, and Supplementary References [file ncomms8115-s1.pdf]

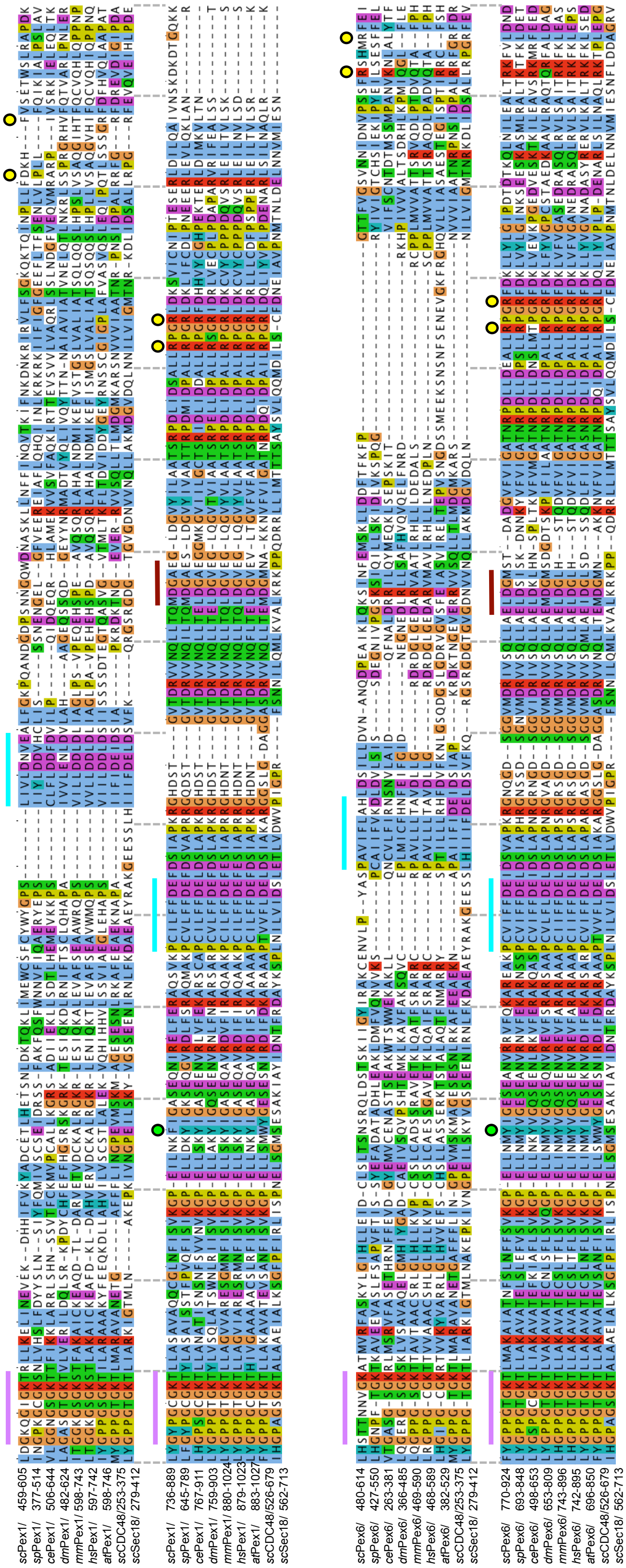

Supplementary Fig. 1: Sequence alignment of selected Pex1/Pex6 homologs.
Sequences of Pex1/6 homologs (sc, Saccharomyces cerevisiae; sp, Schizosaccharomyces pombe; ce Caenorhabditis elegans; dm Drosophila melanogaster; mm, Mus musculus; hs, Homo Sapiens; at, Arabidopsis thaliana), yeast Cdc48 (p97) and Sec18 (NSF) are aligned using ClustalW [1, 2] and visualised with JalView [3]. Canonical AAA+ elements are indicated as follows: Walker A (magenta bar), Walker B (turquois bar), substrate-binding loops (green dots), arginine fingers (yellow dots) and residues within ISS motif (brown bar).

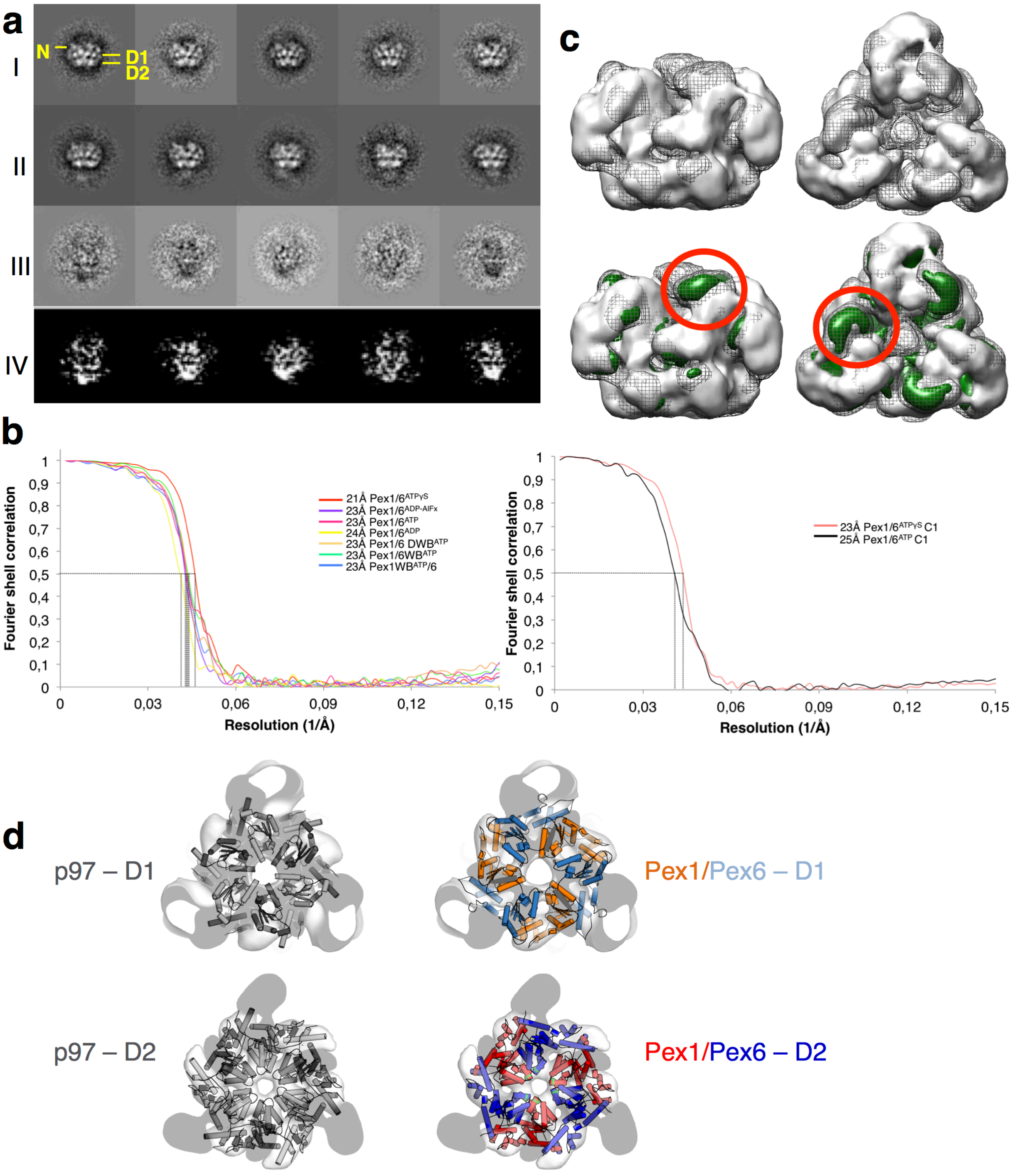

Supplementary Fig. 2: Pex1/Pex6 subunit assignment and rigid body docking.
(a) Side view class averages of Pex1/Pex6 (I) and Pex1GST/Pex6 (II) hexamers assembled in the presence of ATPγS followed by difference images between I and II (III) and binarised difference images (IV). **(b)** Fourier Shell Correlation curves of final 3D reconstructions with C3 symmetry applied and without symmetry (C1), measured at the 0.5 cut-off criterion (colour code and final resolutions are given in inset). The graphs show the Fourier Shell Correlation plotted against spatial frequency [1/Å]. **(c)** Side (left) and top (right) views of surface representations of Δ188Pex1/Pex6^{ATPγS} hexamers (grey surface) overlaid with 3D reconstruction of Pex1/6^{ATPγS} hexamers (dark grey mesh). The difference map between Pex1/6^{ATPγS} and Δ188Pex1/Pex6^{ATPγS} hexamers is shown in green. Difference density that cannot be assigned to domain movements is encircled in red. **(d)** D1 and D2 cross-section views of p97 (pdb-ID: 3CF3, left) and of Pex1/6 homology model (right) automatically docked into the Pex1/6^{ATPγS} EM density map. Pore facing aromatic residues Pex1^{F771} and Pex6^{Y805} are depicted as green spheres.

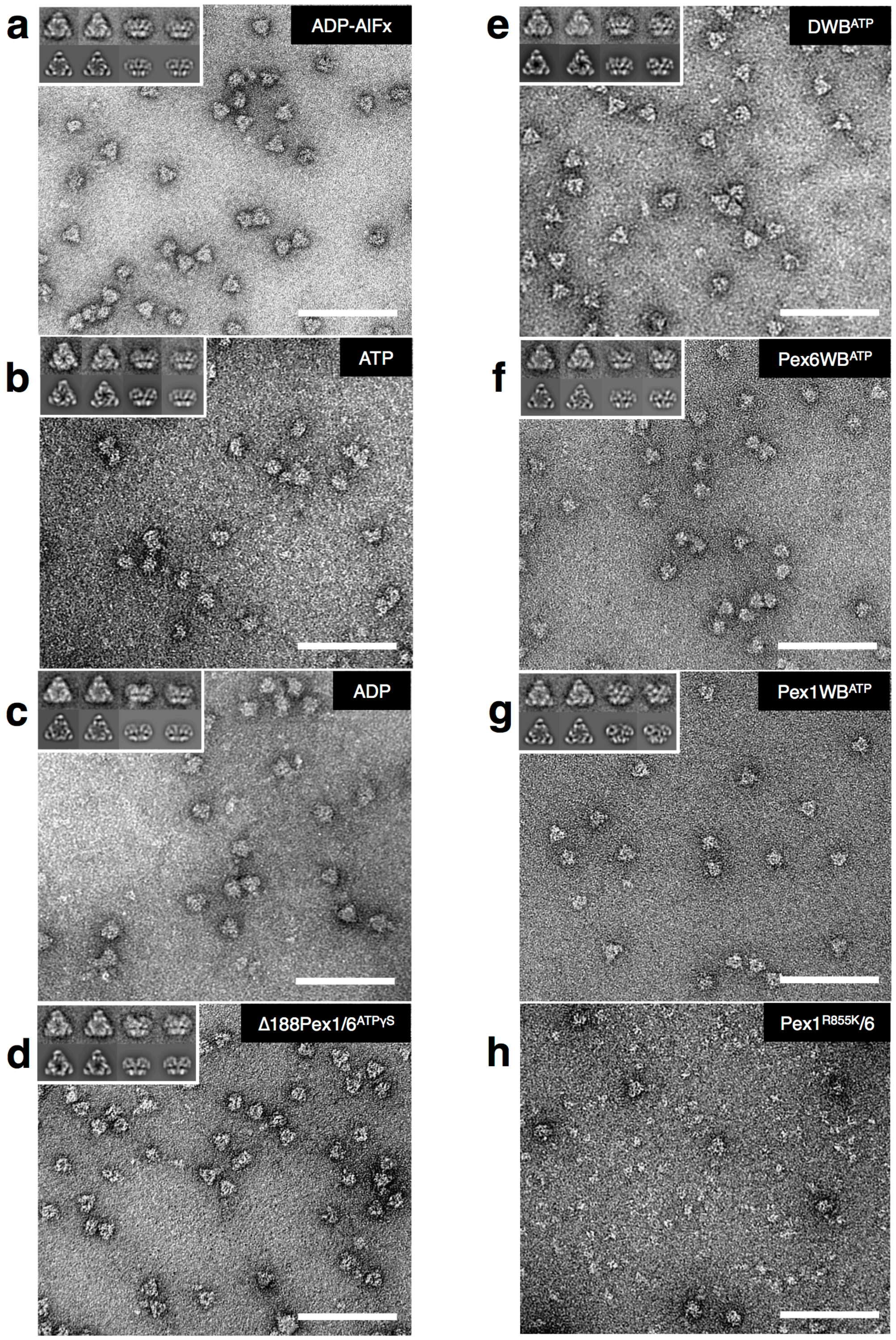

Supplementary Fig. 3: Raw negative stain images of Pex1/6 in the presence of different nucleotides.

Raw electron micrographs of purified Pex1/6 complexes treated with (**a**) ADP-AlFx, (**b**) ATP, (**c**) ADP and (**d**) Δ188Pex1/Pex6 complexes in the presence of ATPγS. ATP was added to Walker B complexes Pex1/6 DWB^{ATP} (**e**), Pex1/6WB^{ATP} (**f**), Pex1WB/6^{ATP} (**g**) and arginine finger variant $Pex1^{R855K}$/6 (**h**). Representative class averages derived from multivariate statistical analysis show characteristic top and side views of Pex1/6 complexes (a-g insets, upper row) and corresponding re-projections of the final 3D reconstruction in the Euler-angle directions assigned to the class averages (a-g insets, lower row). Each class contains an average of five to ten images. Scale bar, 100 nm.

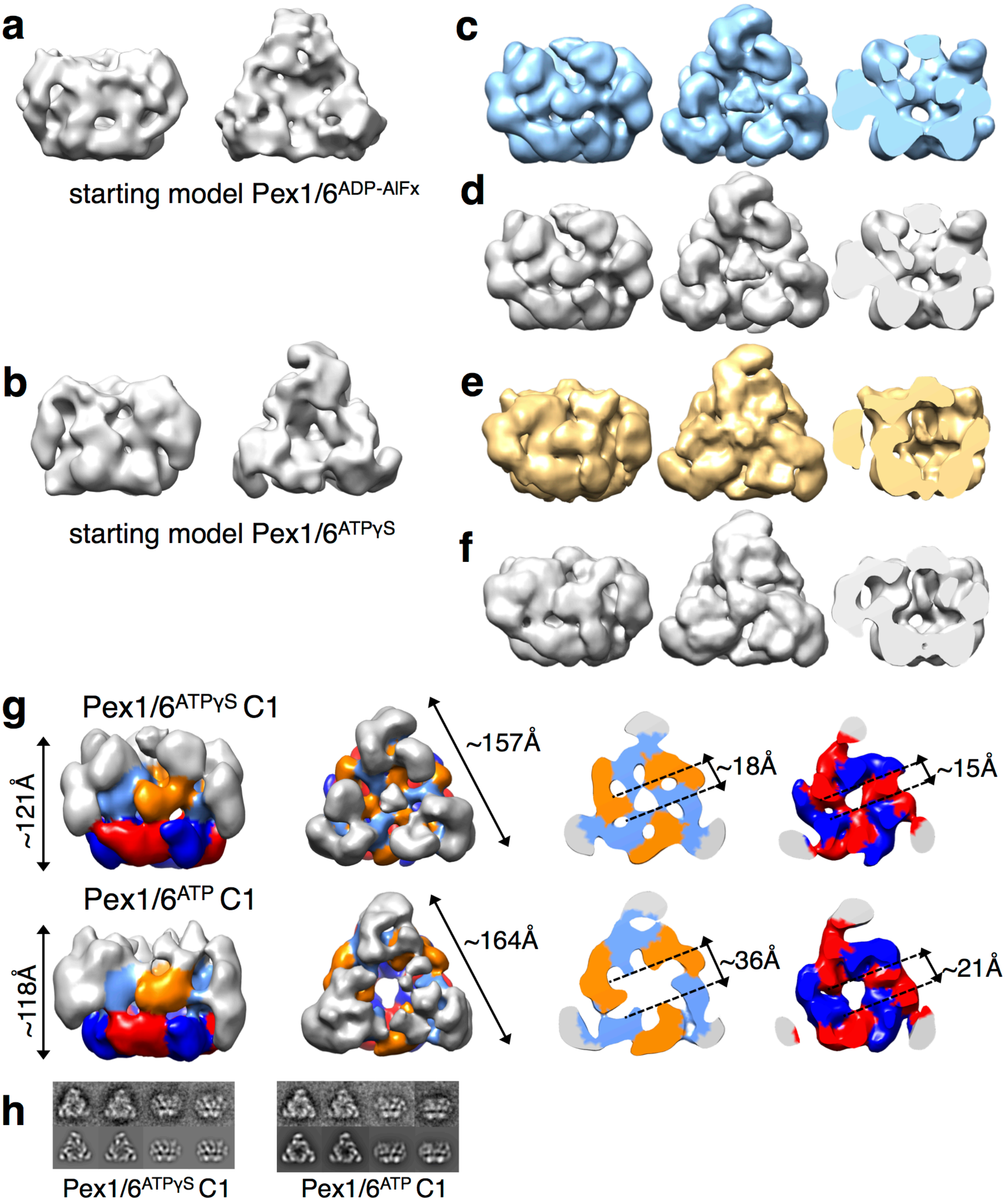

Supplementary Fig. 4: Refinement of Pex1/6$^{ATP\gamma S}$ and Pex1/6$^{ADP\text{-}AlFx}$ from interchanged references and asymmetric Pex1/6 reconstructions.

(a) Top and side view of initial 3D model derived from angular reconstitution of Pex1/6$^{ADP\text{-}AlFx}$ data set. (b) Top and side view of initial 3D model derived from angular reconstitution of Pex1/6$^{ATP\gamma S}$ data set. (c) Final EM reconstruction after projection matching of ATPγS treated single particles to starting model in (a) shown as side, top and side cut open view. (d) Final EM reconstruction after projection matching of ATPγS Pex1/6 data set to starting model in (b) depicted as side, top and side cut open view. (e) Final EM reconstruction after projection matching of ADP-AlFx treated single particles to starting model in (b), shown as side, top and side cut open view. (f) Final EM reconstruction after projection matching of ADP-AlFx Pex1/6 data set to starting model in (a) depicted as side, top and side cut open view. (g) Symmetry free EM reconstructions of Pex1/6$^{ATP\gamma S}$ and Pex1/6^{ATP} complexes as side views, followed by top views and cross-sections of the D1 and D2 layers. (h) Representative class averages derived from multivariate statistical analysis show characteristic top/side views (upper row) and corresponding re-projections (lower row) of the final of 3D reconstructions in the Euler-angle directions assigned to the class averages.

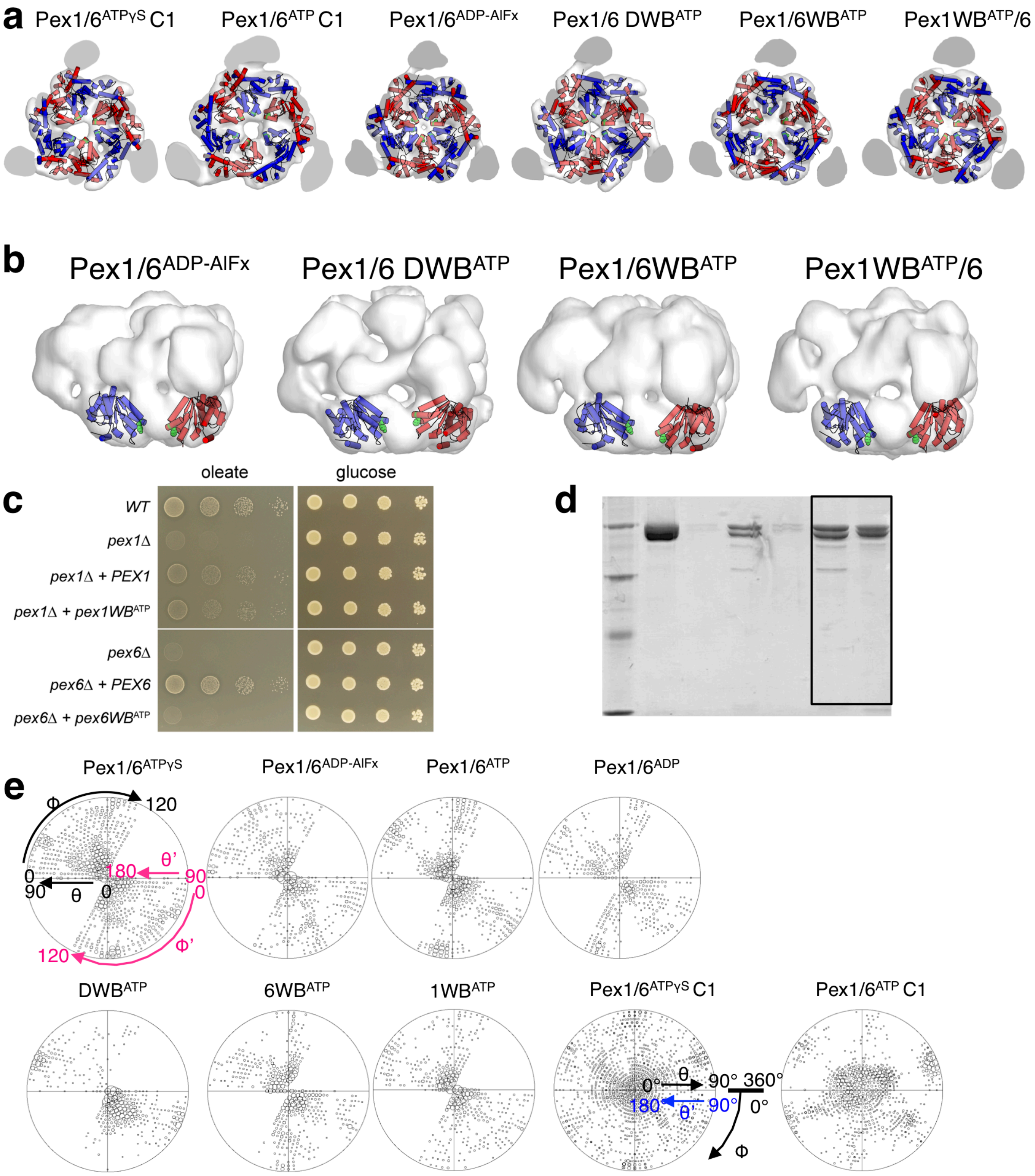

Supplementary Fig. 5: Localisation of substrate binding residues and effects of Pex1/6 Walker B mutants *in vivo.*

(a) Automated rigid body fits of Pex1/6 homology models to EM reconstructions as indicated. (b) Side view surface representation of EM reconstructions as indicated. One Pex1D2 (red) and Pex6D2 (blue) domain of each complex is shown as cartoon representation based on rigid body fitting. Residues $Pex1^{F771}$ and $Pex6^{Y805}$ are depicted as green spheres. (c) Growth of strains expressing either wild type (*PEX1, PEX6*), no (*pex1Δ, pex6Δ*) or mutated Pex1, Pex6 alleles (*pex6*WB^{ATP}, *pex1*WB^{ATP}) on either glucose or oleate as single carbon source. (d) Un-cropped version of the SDS gel shown in main Fig. 1b. Cropped part is indicated. (e) Euler plots for C3 and C1 Pex1/6 datasets after final refinement with projection matching. The polar (θ) and azimuthal (Φ) directions are indicated for $Pex1/6^{ATPγS}$ (directions for mirrored particles, θ' and Φ', are indicated in pink). C3 symmetry axis is placed in the centre of each Euler plot circle. For asymmetric C1 Pex1/6 datasets, respective polar (θ) and azimuthal (Φ) directions are indicated for $Pex1/6^{ATPγS}$ (directions for mirrored particles, θ', are indicated in blue).

Supplementary Table 1: Plasmids used in this study.

Plasmid	Description	Source or reference
pRS425GAL	*GAL1, LEU2, 2µ, Amp*^{r}	gift from C. Enenkel
pYES2	*GAL1, URA3, 2µ, Amp*^{r}	Invitrogen
pRS316	*URA3, CEN/ARS, Amp*^{r}	ATCC® 77145^{TM}
pRS425GAL-*PEX1*-TEV-ProA	*LEU2, 2µ, GAL:PEX1-TEV-ProA*	This study
pRS425GAL-*PEX1*-His6-TEV-ProA	*LEU2, 2µ, GAL:PEX1-His6-TEV-ProA*	This study
pRS425GAL-*PEX1*^{E798Q}-His6-TEV-ProA	*LEU2, 2µ, GAL:PEX1*^{E798Q}*-His*$_6$*-TEV-ProA*	This study
pRS425GAL-Δ188*PEX1*-His6-TEV-ProA	*LEU2, 2µ, GAL:Δ188PEX1-His6-TEV-ProA*	This study
pYES2-*PEX6*-TEV-ProA	*LEU2, 2µ, GAL:PEX6-TEV-ProA*	This study
pYES2-His6-*PEX6*-TEV-ProA	*LEU2, 2µ, GAL:His6-PEX6-TEV-ProA*	This study
pYES2-His6-*PEX6*^{E832Q}-TEV-ProA	*LEU2, 2µ, GAL:*His$_6$-*PEX6*^{E832Q}*-TEV-ProA*	This study
pRS316-p*PEX1*	*URA3, CEN/ARS, PEX1:PEX1*	This study
pRS316-p*PEX6*	*URA3, CEN/ARS, PEX6:PEX6*	This study
pRS316-p*PEX1*^{Y488A}	*URA3, CEN/ARS, PEX1:PEX1*^{Y488A}	This study
pRS316-p*PEX1*^{H495A}	*URA3, CEN/ARS, PEX1:PEX1*^{H495A}	This study
pRS316-p*PEX1*^{F771A}	*URA3, CEN/ARS, PEX1:PEX1*^{F771A}	This study
pRS316-p*PEX1*^{R855K}	*URA3, CEN/ARS, PEX1:PEX1*^{R855K}	This study
pRS316-p*PEX6*^{Y528A}	*URA3, CEN/ARS, PEX6:PEX6*^{Y528A}	This study
pRS316-p*PEX6*^{Y805A}	*URA3, CEN/ARS, PEX6:PEX6*^{Y805A}	This study
pRS316-p*PEX6*^{R607K}	*URA3, CEN/ARS, PEX6:PEX6*^{R607K}	This study
pRS316 -p*PEX6*^{R611K}	*URA3, CEN/ARS, PEX6:PEX6*^{R611K}	This study
pRS316-p*PEX6*^{R892K}	*URA3, CEN/ARS, PEX6:PEX6*^{R892K}	This study
pRS416-p*Pex1*	*URA3, CEN/ARS, PEX1:PEX1*	[4]
pRS416-p*Pex6*	*URA3, CEN/ARS, PEX6:PEX6*	[5]
pRS416-p*Pex1*^{E798Q}	*URA3, CEN/ARS, PEX1:PEX1*^{E798Q}	This study
pRS416-p*Pex6*^{E832Q}	*URA3, CEN/ARS, PEX6:PEX6*^{E832Q}	This study
pRSFDuet-His6-*Pex1*-GST	*Kan*^{R}*, RSF1030, T7:His6-Pex1-GST*	[6]
pRSFDuet-His6-*Pex1*^{E798Q}-GST	*Kan*^{R}*, RSF1030, T7:His6-Pex1*^{E798Q}*-GST*	This study
pRSFDuet-His6-*Pex1*^{R855K}-GST	*Kan*^{R}*, RSF1030, T7:His6-Pex1*^{R855K}*-GST*	This study
pRSFDuet-His6-*Pex1*^{D826V}-GST	*Kan*^{R}*, RSF1030, T7:His6-Pex1*^{D826V}*-GST*	This study
pRSFDuet-His6-*Pex6*	*Kan*^{R}*, RSF1030, T7:His6-Pex6*	[6]
pRSFDuet-His6-*Pex6*^{E832Q}	*Kan*^{R}*, RSF1030, T7:His6-Pex6*^{E832Q}	This study
pRSFDuet-His6-*Pex6*^{R892K}	*Kan*^{R}*, RSF1030, T7:His6-Pex6*^{R892K}	This study

Supplementary Table 2: Primers used in this study.

Primers used for generating PEX1/PEX6 yeast overexpression plasmids

Name	Sequence (5’ -> 3’)
PEX1-fwd	CGAACCAAGCTTATGACGACGACCAAGAGGTTG
ΔR188-PEX1-fwd	AAAAAAGCTTATGCGTTTGGTGAAGGCTGAG
PEX1-rev	GCCGACTCTCCCTTATGGGATCCCAAC
PEX6-fwd	AAAAGGTACCATGAAGGCATCGCTTACGTTT
His$_6$-PEX6-fwd	AAAAGGTACCATGCATCATCATCATCATCATAAGGCATCGCTTACGTTT
PEX6-rev	AAAAGGATCCAGCACCTTCAAAATTAGC
PEX1-D2-E798Q-fwd	CTATTTTTTGACCAATTCGATTCGATCGCGCCA
PEX1-D2-E798Q-rev	TGGCGCGATCGAATCGAATTGGTCAAAAAATAG
PEX6-D2-E832Q-fwd	TGTGTCATATTTTTTGATCAAATCGATTCAGTAGCA
PEX6-D2-E832Q-rev	TGCTACTGAATCGATTTGATCAAAAAATATGACACA

Primers used for generating *PEX1/PEX6 E. coli* overexpression plasmids

**Name**	**Sequence (5' -> 3')**
Throm.cleav.-GST-fwd	GCAAGCTTCCGCGGGGCGGTTCAGGTCTGGTGCCGCGTGGATCTGGCGGTTCAGGTATGTCCCCTATACTAGGT
GST-rev	ATGCGGCCGCTTATGGAGGATGGTCGCCACC
PEX1-fwd	ATAGAGCTCAATGACGACGACCAAGAGGTTGAAG
PEX1-rev	CGCAAGCTTCATAAGGGAGAGTCG
PEX6-fwd	ATTGGATCCCATGAAGGCATCGCTTACGTTTAGTC
PEX6-rev	TATGTCGACTTAAGCACCTTCAAAATTAGCTCTCACC
PEX1-E798Q-fwd	CCTGTATTCTATTTTTTGACCAGTTCGATTCTATTGCGCC
PEX1-E798Q-rev	GGCGCAATAGAATCGAACTGGTCAAAAAATAGAATACAGG
PEX1-R855K-fwd	CGCATTGTTAAGACCGGGAAAATTAGACAAAAGTGTGATCT
PEX1-R855K-rev	AGATCACACTTTTGTCTAATTTTCCCGGTCTTAACAATGCG
PEX1-D826V-fwd	TATTGACCCAAATGGTTGGTGCCGAGGGCC
PEX1-D826V-rev	GGCCCTCGGCACCAACCATTTGGGTCAATA
PEX6-E832Q-fwd	CTTGTGTCATATTTTTCGATCAAATCGATTCAGTAGCACCC
PEX6-E832Q-rev	GGGTGCTACTGAATCGATTTGATCGAAAAATATGACACAAG
PEX6-R892K-fwd	GACGAAGCACTACTAAGACCAGGAAAATTCGATAAATTGTTATATTTAGGC
PEX6-R892K-rev	GCCTAAATATAACAATTTATCGAATTTTCCTGGTCTTAGTAGTGCTTCGTC

Primers used for generating *PEX1/PEX6* plasmids for oleate growth assays

**Name**	**Sequence (5' -> 3')**
Prom-PEX1-fwd	AAAATCTAGATAATTCTATGTAACCTCGATGGC
Prom-PEX1-rev	AAAACTCGAGTCAGGATCCCATAAGGGAGAGTCGGCTACCAAT
Prom-PEX6-fwd	AAAATCTAGAAAGAACTTTATATATCATGTAGC
Prom-PEX6-rev	AAAAGGTACCTTAAGCACCTTCAAAATTAGC
PEX1-D1-Y488A-fwd	CACATCTTCGTTAAAGCTGCAGATTGTGAAACATTGC
PEX1-D1-Y488A-rev	GCAATGTTTCACAATCTGCAGCTTTAACGAAGATGTG
PEX1-D1-H495A-fwd	GCGGATTGTGAAACGCTAGCTGAGACATCAAATTTA
PEX1-D1-H495A-rev	TAAATTTGATGTCTCAGCTAGCGTTTCACAATCCGC
PEX1-D2-F771A-fwd	CAGAGATTTTAAACAAGGCGATCGGTGCCAGCGAACA
PEX1-D2-F771A-rev	TGTTCGCTGGCACCGATCGCCTTGTTTAAAATCTCTG
PEX1-D2-R855K-fwd	TTAAGACCGGGAAAGCTTGACAAAAGTGTGATCTGT
PEX1-D2-R855K-rev	ACAGATCACACTTTTGTCAAGCTTTCCCGGTCTTAA
PEX6-D1-Y528A-fwd	ACATCTAAGATTATTGGCGCCATTAGGGCTAAATGTG
PEX6-D1-Y528A-rev	CACATTTAGCCCTAATGGCGCCAATAATCTTAGATGT
PEX6-D1-R607K-fwd	GATAACGTGCCCTCGAGCTTTAAATCACATATGAGA
PEX6-D1-R607K-rev	TCTCATATGTGATTTAAAGCTCGAGGGCACGTT ATC
PEX6-D1-R611K-fwd	GATCACATATGAAATTTGAGATCTTAGTACCCGTTC
PEX6-D1-R611K-rev	GAACGGGTACTAAGATCTCAAATTTCATATGTGATC
PEX6-D2-Y805A-fwd	GAACTGTTGAATATGGCGATCGGTGAGAGTGAAGCTA
PEX6-D2-Y805A-rev	TAGCTTCACTCTCACCGATCGCCATATTCAACAGTTC
PEX6-D2-R892K-fwd	GCACTACTAAGCCCGGGAAAATTCGATAAATTGTTA
PEX6-D2-R892K-rev	TAACAATTTATCGAATTTTCCCGGGCTTAGTAGTGC
PEX1-D2-E798Q-fwd	CCTGTATTCTATTTTTTGACCAGTTCGATTCTATTGCGCC
PEX1-D2-E798Q-rev	GGCGCAATAGAATCGAACTGGTCAAAAAATAGAATACAGG
PEX6-D2-E832Q-fwd	CTTGTGTCATATTTTTCGATCAAATCGATTCAGTAGCACCC
PEX6-D2-E832Q-rev	GGGTGCTACTGAATCGATTTGATCGAAAAATATGACACAAG

Supplementary Table 3: Yeast strains used in this study for protein expression in *S. cerevisiae*.

S. cerevisiae **strain**	**Genotype**	**Source or reference**
WCGa	*MATa his3-11,15leu2-3, 112 ura3 can GAL*	[7]
Pex1-TEV-ProA + His6-*pex6*^{E832Q}-TEV-ProA	*MATa his3-11,15leu2-3, 112 ura3 can GAL* [pRS425GAL-*PEX1*-TEV-ProA], [pYES2-His6-*PEX6*^{E832Q}-TEV-ProA]	This study
pex1^{E798Q}-His6-TEV-ProA + Pex6-TEV-ProA	*MATa his3-11,15leu2-3, 112 ura3 can GAL* [pRS425GAL-*PEX1*^{E798Q}-His6-TEV-ProA], [pYES2-*PEX6*-TEV-ProA]	This study
pex1^{E798Q}-His6-TEV-ProA + His6-*pex6*^{E832Q}-TEV-ProA	*MATa his3-11,15leu2-3, 112 ura3 can GAL* [pRS425GAL-*PEX1*^{E798Q}-His6-TEV-ProA], [pYES2-His6-*PEX6*^{E832Q}-TEV-ProA]	This study
Δ188*pex1*-His6-TEV-ProA+ Pex6-TEV-ProA	*MATa his3-11,15leu2-3, 112 ura3 can GAL* [pRS425GAL-Δ188*PEX1*-His6-TEV-ProA], [pYES2-*PEX6*-TEV-ProA]	This study

Supplementary Table 4: Yeast strains used in this study for oleate growth assays.

S. cerevisiae **strain**	**Genotype**	**Source or reference**
BY 4742	*MATa ; his3D1; leu2D0; lys2D0; ura3D0*	Euroscarf
BY 4742Δ*pex1*	*MATa; his3D1; leu2D0; lys2D0; ura3D0; YKL197c::kanMX4*	Euroscarf
BY 4742Δ*pex6*	*MATa; his3D1; leu2D0; lys2D0; ura3D0; YNL329c::kanMX4*	Euroscarf
Δ*pex1* + pPex1	*MATa; his3D1; leu2D0; lys2D0; ura3D0; YKL197c::kanMX4* ,[pRS316-p*PEX1]*	This study
Δ*pex1* + p*pex1*^{Y488A}	*MATa; his3D1; leu2D0; lys2D0; ura3D0; YKL197c::kanMX4* ,[pRS316-p*PEX1*^{Y488A}*]*	This study
Δ*pex1* + p*pex1*^{H495A}	*MATa; his3D1; leu2D0; lys2D0; ura3D0; YKL197c::kanMX4,* [pRS316-p*PEX1*^{H495A}*]*	This study
Δ*pex1* + p*pex1*^{F771A}	*MATa; his3D1; leu2D0; lys2D0; ura3D0; YKL197c::kanMX4,* [pRS316-p*PEX1*^{F771A}*]*	This study
Δ*pex1* + p*pex1*^{R855K}	*MATa; his3D1; leu2D0; lys2D0; ura3D0; YKL197c::kanMX4,* [pRS316-p*PEX1*^{R855K}*]*	This study
Δ*pex6* +pPex6	*MATa; his3D1; leu2D0; lys2D0; ura3D0; YNL329c::kanMX4*, [pRS316-p*PEX6*]	This study
Δ*pex6* +p*pex6*^{Y528A}	*MATa; his3D1; leu2D0; lys2D0; ura3D0; YNL329c::kanMX4*, [pRS316-p*PEX6*^{Y528A}]	This study
Δ*pex6* +p*pex6*^{Y805A}	*MATa; his3D1; leu2D0; lys2D0; ura3D0; YNL329c::kanMX4*, [pRS316-p*PEX6*^{Y805A}]	This study
Δ*pex6* +p*pex6*^{R607K}	*MATa; his3D1; leu2D0; lys2D0; ura3D0; YNL329c::kanMX4*, [pRS316-p*PEX6*^{R607K}]	This study
Δ*pex6* +p*pex6*^{R611K}	*MATa; his3D1; leu2D0; lys2D0; ura3D0; YNL329c::kanMX4*, [pRS316 -p*PEX6*^{R611K}]	This study
Δ*pex6* +p*pex6*^{R892K}	*MATa; his3D1; leu2D0; lys2D0; ura3D0; YNL329c::kanMX4*, [pRS316-p*PEX6*^{R892K}]	This study
UTL-7A	*MATa; ura3-52; trp1; leu2-3/ 112*	W. Duntze, Bochum
UTL-7AΔ*pex1*	*MATa; ura3-52; trp1; leu2-3/ 112;pex1::loxP*	[8]
UTL-7AΔ*pex6*	*MATa; ura3-52; trp1; leu2-3/ 112;pex6::leu2*	[8]
Δ*pex1* +p*Pex1*	*MATa; ura3-52; trp1; leu2-3/ 112;pex1::loxP,* [pRS416-p*Pex1*]	This study
Δ*pex6* +p*Pex6*	*MATa; ura3-52; trp1; leu2-3/ 112;pex6:: leu2,* [pRS416-p*Pex6*]	This study
Δ*pex1* +p*Pex1*^{E798Q}	*MATa; ura3-52; trp1; leu2-3/ 112;pex1::loxP,* [pRS416-p*Pex1*^{E798Q}]	This study
Δ*pex6* +p*Pex6*^{E832Q}	*MATa; ura3-52; trp1; leu2-3/ 112;pex6:: leu2,* [pRS416-p*Pex6*^{E832Q}]	This study

Supplementary References

1. Goujon M*, et al.* A new bioinformatics analysis tools framework at EMBL-EBI. *Nucleic Acids Res* **38**, W695-699 (2010).
2. Larkin MA*, et al.* ClustalW and ClustalX version 2. *Bioinformatics* **23**, 2947-2948 (2007).
3. Waterhouse AM, Procter JB, Martin DM, Clamp M, Barton GJ. Jalview Version 2--a multiple sequence alignment editor and analysis workbench. *Bioinformatics* **25**, 1189-1191 (2009).
4. Birschmann I*, et al.* Pex15p of Saccharomyces cerevisiae provides a molecular basis for recruitment of the AAA peroxin Pex6p to peroxisomal membranes. *Mol Biol Cell* **14**, 2226-2236 (2003).
5. Birschmann I, Rosenkranz K, Erdmann R, Kunau WH. Structural and functional analysis of the interaction of the AAA-peroxins Pex1p and Pex6p. *FEBS J* **272**, 47-58 (2005).
6. Saffian D, Grimm I, Girzalsky W, Erdmann R. ATP-dependent assembly of the heteromeric Pex1p-Pex6p-complex of the peroxisomal matrix protein import machinery. *J Struct Biol* **179**, 126-132 (2012).
7. Heinemeyer W, Trondle N, Albrecht G, Wolf DH. PRE5 and PRE6, the last missing genes encoding 20S proteasome subunits from yeast? Indication for a set of 14 different subunits in the eukaryotic proteasome core. *Biochemistry* **33**, 12229-12237 (1994).
8. Platta HW, Girzalsky W, Erdmann R. Ubiquitination of the peroxisomal import receptor Pex5p. *Biochem J* **384**, 37-45 (2004).
